# The effect of tuberculosis on immune reconstitution among HIV patients on highly active antiretroviral therapy in Adigrat general hospital, eastern Tigrai, Ethiopia; 2019: a retrospective follow up study

**DOI:** 10.1186/s12865-019-0327-7

**Published:** 2019-12-05

**Authors:** Hadush Negash, Haftom Legese, Mebrahtu Tefera, Fitsum Mardu, Kebede Tesfay, Senait Gebresilasie, Berhane Fseha, Tsega Kahsay, Aderajew Gebrewahd, Brhane Berhe

**Affiliations:** 1 Unit of Medical Microbiology, Department of Medical Laboratory Sciences, College of Medicine and Health Sciences, Adigrat University, Tigrai, Ethiopia; 2 Unit of Quality assurance, Department of Medical Laboratory Sciences, College of Medicine and Health Sciences, Adigrat University, Tigrai, Ethiopia; 3 Unit of Medical Parasitology, Department of Medical Laboratory Sciences, College of Medicine and Health Sciences, Adigrat University, Tigrai, Ethiopia; 4 Unit of Pediatrics and child health, Department of Midwifery, College of Medicine and Health Sciences, Adigrat University, Tigrai, Ethiopia; 5 Department of Public Health, College of Medicine and Health Science, Adigrat University, Adigrat, Ethiopia

**Keywords:** Adigrat general hospital, HAART, HIV patients, Immunological response, Retrospective follow up, Tuberculosis

## Abstract

**Background:**

Ethiopia initiated antiretroviral therapy early in 2005. Managing and detecting antiretroviral treatment response is important to monitor the effectiveness of medication and possible drug switching for low immune reconstitution. There is less recovery of CD4+ T cells among human immunodeficiency virus patients infected with tuberculosis. Hence, we aimed to assess the effect of tuberculosis and other determinant factors of immunological response among human immunodeficiency virus patients on highly active antiretroviral therapy. A retrospective follow up study was conducted from October to July 2019. A total of 393 participants were enrolled. An interviewer based questionnaire was used for data collection. Patient charts were used to extract clinical data and follow up results of the CD4+ T cell. Current CD4+ T cell counts of patients were performed. STATA 13 software was used to analyze the data. A *p*-value ≤0.05 was considered a statistically significant association.

**Results:**

The mean age of study participants was 39.2 years (SD: + 12.2 years) with 8.32 mean years of follow up. The overall prevalence of immune reconstitution failure was 24.7% (97/393). Highest failure rate occurred within the first year of follow up time, 15.7 per 100 Person-year. Failure of CD4+ T cells reconstitution was higher among tuberculosis coinfected patients (48.8%) than mono-infected patients (13.7%). Living in an urban residence, baseline CD4+ T cell count ≤250 cells/mm^3^, poor treatment adherence and tuberculosis infection were significantly associated with the immunological failure.

**Conclusions:**

There was a high rate of CD4+ T cells reconstitution failure among our study participants. Tuberculosis infection increased the rate of failure. Factors like low CD4+ T cell baseline count, poor adherence and urban residence were associated with the immunological failure. There should be strict monitoring of CD4+ T cell counts among individuals with tuberculosis coinfection.

## Background

Human Immunodeficiency Virus (HIV) infection is still a major public health problem in the globe. In countries of Sub-Sahara, there were about 23 million HIV infected people. Most of the infected Sub-Saharan patients had reported late to health facilities for treatment [1, 2]. HIV infection depletes immune cells especially CD4+ T lymphocytes [3]. Immune destruction due to HIV proceeds increased morbidity and mortality which may also be associated with different opportunistic infections and tuberculosis (TB) [4].

Globally, by the year 2017, there were about 20.9 million HIV patients estimated to receive highly active antiretroviral therapy (HAART) [5]. Antiretroviral drugs are targeted at inhibiting viral attachment and replication. Hence, leading to the recovery of immune function [3, 6]. HAART reduces HIV associated morbidity and mortality which in turn enables patients to have a productive life and stay longer. CD4+ T cell count is one of the important markers for assessing treatment response and immune recovery among HIV patients on HAART. World health organization (WHO) recommends that CD4+ T cell count should be performed before HAART, at 3, 6, 9 and 12 months after initiation of HAART. Determine the CD4+ cell counts at every follow-up time helps clinicians to confirm suspected treatment failure detected clinically and immunologically to provide necessary interventions (adherence support and HAART regimen switching) [6].

Among HIV infected patients in developing countries, TB remains a major public health problem [7]. Moreover, HIV is the most potent risk factor for TB. Additionally, TB is the leading cause of morbidity and mortality in HIV infected patients [8]. Among peoples of African countries, the prevalence of immunological failure to HAART medication is increased [9]. HIV/TB co-infected patients are less likely to have good recovery of CD4+ cells. However, studies assessing CD4+ T cells reconstitution of individuals on HAART have found to be poor CD4+ T cells recovery occured among patients who developed TB after initiating therapy [10, 11]. TB infection can also cause T lymphocytopenia in which the consequence is worsened due to HIV co-infection [12].

In Ethiopia, access to HAART started massively in 2005 [13]. The treatment has been successful as demonstrated by the improvement of survival and immune recovery [14, 15]. This makes HIV a manageable disease. Some studies indicated the level of adherence is suboptimal for patients on HAART ranging from 7 to 28% [16–21]. Generally, the longer a person is failing HAART, the higher the mortality [22]. Monitoring immunological treatment response of HAART is challenging in resource-limited settings. Regular follow up of HAART among HIV patients detection and management of treatment failure is recommended [23, 24]. Although TB/HIV coinfection is a major public health problem in Ethiopia, there are few studies that have reported the effect of TB on immunological responses of HIV patients during HAART. Hence, assessing the effect of TB and other determinant factors on immune reconstitution of HIV patients will provide information for clinicians for appropriate management of TB/HIV co-infected patients.

## Methods

### Study design, area and period

A retrospective follow up study was employed to assess the effect of TB and other determinant factors of immune reconstitution among HIV patients on HAART in Adigrat General Hospital, Eastern Tigrai, Ethiopia from October to July 2019. Adigrat is a zonal administrative town of Eastern Tigrai, with an estimated population of 76,400 [25]. The town is located at a longitude and latitude 14°16′N 39°27′E, with an elevation of 2457 m (8,061 ft) above sea level and below a high ridge to the west.

Adigrat General Hospital is one of the governmental Hospitals located in Adigrat established for peoples from 6 weredas (districts). The Hospital is currently involved as teaching and referral services for more than 1,000,000 population with an average annual client flow of 131,125 people. The Hospital has about 120 beds with a total of 209 health care providers and 132 administrative staffs. There are about 10 health care providers in the HAART clinic of the Hospital and a total of 1582 HIV patients currently on HAART follow up.

### Source population and study population

- All HIV patients who were currently on HAART and screened for TB infection during or after initiation of the HAART

### Sampling technique and sample size

- Simple random sampling technique was used to enroll the study participants from the sample frame of HIV patients list in the HAART clinic.
- Sample size was calculated using double population proportion and determined by Epi info version 7.1 software comparing the proportion of treatment response among TB exposed and non-exposed groups of the population and using 80% power.
- 393 HIV patients were included with the ratio of 1:2 (123 exposed to TB and 270 non-exposed to TB).

### Eligibility criteria

#### Inclusion criteria

- HIV patients complete charts and with at least 6 months of follow up of HAART
- HIV patients who were transferredin with full previous data and initial baseline CD4+ count
- HIV patients who have provided written informed consent or assent to participate in the study

#### Exclusion criteria

- HIV patients who were lost to follow up to course of HAART

### Data collection, preparation and analysis

#### Data collection

An interviewer based semi structured questionnaire with some open and closed ended questions was used to collect socio-demographic data (age, sex, modern contraceptive intake and residence). Clinical data (HAART start date, duration of ART, adherence, TB co-infection, BMI, eligibility criteria, Cotrimoxazole intake, functional status, WHO stage, status of TB and presence of opportunistic infections) and previous follow up results of CD4 + T cells (baseline CD4 + cell count, and follow up CD4+ T cell counts) from patients’ medical records and charts.

#### Sample collection

About 2 ml of venous blood was collected from each patient by trained laboratory professionals. Current CD4+ cell counts were performed using BD FACS Presto™ cartridge machine (BD Becton, Dickson, BD Biosciences, New Zealand). We performed the CD4+ T cell counts using BD FACS Presto machine which is used to determine CD4+ T cells, CD4+ percentage and hemoglobin of the HIV patients on HAART. The cartridge of the BD FACS Presto contains dried fluorochrome-conjugated antibody reagents wherby bind with the CD4+ T cells. CD4+ T cell will be counted, when blood reacts with the dried fluorochrome-conjugated antibodies, the antibodies bind to the surface antigens on the T lymphocytes. After 18 min of incubation, the cells are analyzed on the BD FACS Presto™ near-patient CD4+ T cell counter.

### Data analysis

We used STATA version 13 software for data entry and analysis. Descriptive statistics were performed. Data were summarized and organized using figures and frequency tables. Bi-variate and multi-variate regression analysis were employed to measure the association between dependent and independent variables. Variables with *p* <  0.20 in the bivariate logistic regression were transferred to multi-variate regression analysis to identify the factors that have statistical significance and significantly associated with the dependent variable. A *p*-value of ≤0.05 was considered as statistical significant.

### Operational definition

#### Treatment adherence

Taking HAART medications properly on a consistent basis over time and not missing doses of the prescribed therapeutic agents [6].

- **Good adherence:** When the average treatment adherence of HIV patients is greater than or equal to 95%.
- **Average adherence:** The average treatment adherence by the patients is from 85 to 94%.
- **Weak adherence:** The average treatment adherence by the patients is less than 85%.

#### Immune reconstitution

The ability of the body to respond to HAART and recovery of CD4+ T cell counts

- **Failure in immune reconstitution:** CD4+ T cell counts to baseline or below/CD4+ T cell counts below 100 cells/mm3 for consecutive tests during the course of HAART [6].
- **Success in immune reconstitution**: CD4+ T cell counts greater than the baseline count after initiation of HAART [6].

## Results

### Socio-demographic characteristics of participants

A total of 393 HIV patients enrolled in HAART were included in our study. Out of the total, 262 (66.7%) were females. The mean age of the study participants was 39.2 years (ranging from 5 to 78 years; SD of 12.2 years). The HIV patients were on follow up from 6 to 180 months with a median of 121(IQR; 66–132) months. Most of the study participants were from urban residents, 249 (63.4%) (Table 1).

**Table 1:** Bivariate and multivariate analysis of determinant factors with immune reconstitution of HAART among HIV patients in Adigrat General Hospital, Eastern Tigrai, Ethiopia; 2019: A retrospective follow up (*n* = 393)

Variables	Category	Immune reconstitution		*P* value	COR	95% CI	*P* value	AOR	95%CI
		Failure N [%]	Success, N [%]						
Sex	Male	38 [29]	93 [71]		1.3	[0.156–2.026	0.149	1.6	[0.840–3.152]
	Female	59 [22.5]	203 [77.5]	0.160	1.0			1.0	
Age category	< 15 years	13 [59]	09 [41]	0.001	7.2	[2.561–16.524]	0.921	2.3	[0.151–5.523]
	15–30 years	17 [35.4]	31 [64.6]	0.002	2.7	[1.310–4.374]	0.819	1.4	[0.023–2.391]
	31–45 years	48 [23.4]	157 [76.6]	0.019	1.5	[0.166–2.266]	0.648	1.2	[0.062–3.527]
	46–60 years	17 [16]	89 [84]	0.362	0.9	[0.712–2.531]	0.173	0.6	[0.242–1.291]
	> 60 years	02 [16.7]	10 [83.3]		1.0			1.0	
Residence	Urban	73 [29.3]	176 [70.7]	0.006	2.1	[1.238–3.475]	0.020*	2.3	[1.137–4.602]
	Rural	24 [16.7]	120 [83.3]		1.0			1.0	
BMI	Normal	46 [20.5]	178 [79.5]		1.0			1.0	
	Overweight	06 [16.2]	31 [83.8]	0.010	0.75	[0.071–2.638]	0.203	1.5	[0.793–2.976]
	Undernourished	45 [34]	87 [66]	0.000	2.0	[0.232–3.674]	0.647	1.2	[0.364–1.873]
Cotrimoxazole intake	No	23 [28.7]	57 [71.3]	0.345	1.3	[0.443–1.930]			
	Yes	74 [23.6]	239 [76.4]		1.0				
Eligibility for HAART	Clinical staging	78 [24.8]	236 [75.2]		1.0			1.0	
	Test and treat	09 [37.5]	15 [62.5]	0.056	1.8	[0.243–3.378]	0.074	0.8	[0.153–1.090
	Transferred in	10 [18.2]	45 [81.8]	0.080	0.7	[0.089–1.409]	0.647	1.09	[0.364–1.873]
Functional status	Working	89 [24.5]	275 [75.5]		1.0				
	Ambulatory	7 [26.9]	19 [73.1]	0.288	1.1	[0.119–2.780]			
	Bedridden	01 [33.3]	02 [66.7]	0.231	1.5	[0.038–3.987]			
Baseline CD4+ T cell counts	> 250	39 [16.1]	203 [83.9]		1.0			1.0	
	≤ 250	58 [38.4]	93 [61.6]	< 0.001	3.2	[1.342–5.445]	< 0.001*	4.2	[2.977–7.961]
WHO stage	Stage I	83 [23.7]	267 [76.3]		1.0			1.0	
	Stage II	05 [23.8]	16 [76.2]	0.022	0.99	[0.234–1.871]	0.819	1.2	[0.396–3.223]
	Stage III	08 [47.1]	09 [52.9]	0.151	2.9	[0.293–5.209]	0.648	1.5	[0.268–8.318]
	Stage IV	01 [20]	04 [80]	0.091	0.8	[0.198–3.783]	0.798	0.7	[0.027–16.329]
Modern non-hormonal contraceptive (*n* = 262)	No	34 [22.5]	117 [77.5]		1.0				
	Yes	31 [27.9]	80 [72.1]	0.234	1.3	[0.834–2.098]			
Treatment adherence	Good	41 [14.4]	244 [85.6]		1.0			1.0	
	Average	14 [46.7]	16 [53.3]	0.034	5.2	[2.573–8.101]	0.107	5.6	[2.978–9.829]
	Weak	42 [53.8]	36 [46.2]	< 0.001	7.0	[3.987–12.090]	< 0.001*	9.4	[4.497–19.700]
Opportunistic infections	Yes	51 [26.3]	143 [73.7]	0.466	1.2	[0.749–1.877]			
	No	46 [23.1]	153 [76.9]		1.0			1.0	
Status TB	Positive	60 [48.8]	63 [51.2]	< 0.001	6.0	[3.655–9.842]	< 0.001*	11.5	[5.704–23.191]
	Negative	37 [13.7]	233 [86.3]		1.0			1.0	
Duration of HAART	≤ 2 years	12 [35.3]	22 [64.7]		1.0				
	2–5 years	26 [26.3]	73 [73.7]	0.314	0.9	[0.250–1.561]			
	5–10 years	15 [25.4]	44 [74.6]	0.907	1.1	[0.458–2.001]			
	> 10 years	44 [21.9]	157 [78.1]	0.569	1.3	[0.620–2.388]			

Key: “*” indicates a significant association, *AOR* Adjusted odds ratio, *CI* Confidence interval, *COR* Crude odds ratio

### Baseline clinical and laboratory characteristics of participants

The median baseline CD4+ T cell count of the study participants on HAART was 196 cells/μl ranging from 5 to 1315 cells/μl (IQR; 112–316 cells/μl). The mean baseline CD4+ T cell counts among HIV and HIV/TB infected individuals was 240 and 258 cells/μl. The HAART enrollment eligibility criteria for most of the study participants, 314 (79.9%) was due to clinical staging. One hundred twenty-three (31.3%) of the total study participants developed active TB within the 180 months of follow up with an incident of 4.04 per 100 PY. Most of the study participants, 350 (89.1%) were in WHO clinical stage I (Table 2).

**Table 2:** Demographic, laboratory and clinical characterstics of HIV patients on HAART in Adigrat General Hospital, Eastern Tigrai, Ethiopia; 2019: A retrospective follow up (*n* = 393)

Variables	Category	Frequency, *N* [%]	TB status of HIV patients	
			Negative, *N* [%]	Positive, *N* [%]
Sex	Male	131 [33.3]	87 [66.4]	44 [33.6]
	Female	262 [66.7]	183 [69.8]	79 [30.2]
Age category	< 15 years	22 [5.6]	16 [72.7]	06 [27.3]
	15–30 years	48 [12.2]	38 [79.2]	10 [20.8]
	31–45 years	205 [52.2]	131 [63.9]	74 [36.1]
	46–60 years	106 [27]	77 [72.6]	29 [27.4]
	> 60 years	12 [3.1]	08 [66.7]	04 [33.3]
Residence	Urban	249 [63.4]	168 [67.5]	81 [32.5]
	Rural	144 [36.6]	102 [70.8]	42 [29.2]
BMI	Normal	224 [57]	155 [69.2]	69 [30.8]
	Overweight	37 [9.4]	32 [86.5]	05 [13.5]
	Undernourished	132 [33.6]	83 [62.9]	49 [37.1]
Cotrimoxazole intake	No	80 [20.4]	53 [66.3]	27 [33.7]
	Yes	313 [79.6]	217 [69.3]	96 [29.7]
Eligibility criteria	Clinical staging	314 [79.9]	222 [70.7]	92 [29.3]
	Test and treat	24 [6.1]	13 [54.2]	11 [45.8]
	Transferred in	55 [14]	35 [63.6]	20 [36.4]
Functional status	Working	364 [92.6]	246 [67.6]	118 [32.4]
	Ambulatory	26 [6.6]	23 [88.5]	03 [11.5]
	Bedridden	3 [0.8]	01 [33.3]	02 [66.7]
WHO stage	Stage I	350 [89.1]	243 [69.4]	107 [30.6]
	Stage II	21 [5.3]	17 [81.0]	04 [19.0]
	Stage III	17 [4.3]	07 [41.2]	10 [58.8]
	Stage IV	5 [1.3]	03 [60.0]	02 [40.0]
Modern non-hormonal contraceptive (n = 262)	No	151 [57.6]	109 [72.2]	42 [27.8]
	Yes	111 [42.4]	74 [66.7]	37 [33.3]
Treatment adherence	Good	285 [72.5]	203 [71.2]	48 [28.8]
	Average	30 [7.6]	19 [63.3]	11 [36.7]
	Weak	78 [19.9]	48 [61.5]	30 [38.5]
Opportunistic infections	Yes	194 [49.4]	132 [68.0]	62 [32.0]
	No	199 [50.6]	138 [69.3]	61 [30.7]
HAART regimen	1a	25 [6.4]	19 [76.0]	06 [24.0]
	1c	152 [38.7]	109 [71.7]	43 [28.3]
	1d	52 [13.2]	37 [71.2]	15 [28.8]
	1e	122 [31.0]	77 [63.1]	45 [36.9]
	1f	42 [10.7]	28 [66.7]	14 [33.3]
Baseline CD4+ T cell counts	> 250	242 [61.6]	168 [64.1]	74 [35.9]
	≤ 250	151 [38.4]	102 [67.5]	49 [32.5]
Average baseline CD4+ T cell counts			240 cells/μl	258 cells/μl
Average current CD4+ T cell counts			528 cells/μl	414 cells/μl
Duration of HAART	≤ 2 years	59 [15.0]	38 [64.4]	21 [35.6]
	2–5 years	34 [8.7]	23 [67.6]	11 [33.4]
	5–10 years	99 [25.2]	69 [69.7]	30 [30.3]
	> 10 years	201 [51.1]	140 [69.7]	61 [30.3]

Keys; 1a: d4T + 3TC + NVP; 1c: AZT + 3TC + NVP; 1d: AZT + 3TC + EFV; 1e: TDF + 3TC+ EFV; 1f: TDF + 3TC + NVP

The overall failure of immune reconstitution of HIV patients in our study was 97 (24.7%). About 62 (63.9%) of all the immune reconstitution failures of CD4+ T cell recovery had occurred within 1 year of HAART follow up. The rate of immune failure from our study participants on follow up was 15.78 per 100 PY at the end of 12 months, 8.27 per 100 PY at the end of 24 months, 6.11 per 100 PY at the end of 36 months, 4.32 per 100 PY at the end of 60 months and 2.47 per 100 PY at the end of 120 months.

### Effect of TB on the immune reconstitution of participants

**Fig. 1:**
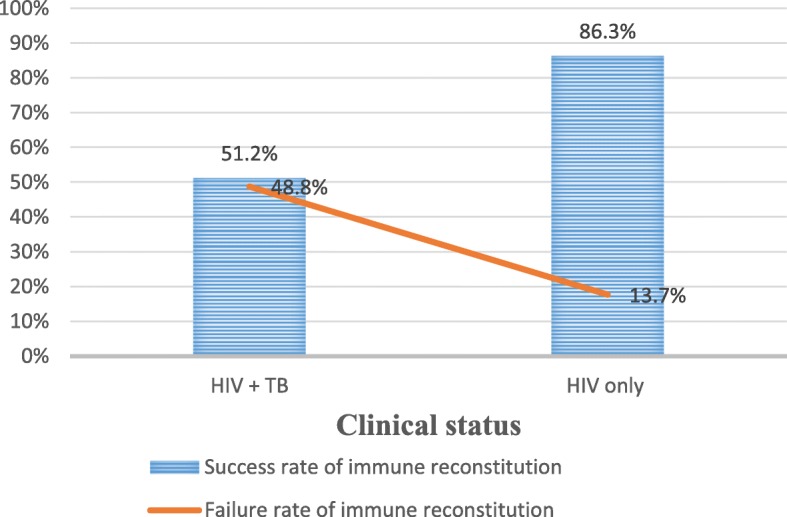
The effect of tuberculosis on the immune reconstitution among HIV patients on HAART in Adigrat General Hospital, Eastern Tigrai, Ethiopia; 2019: A retrospective follow up study (*n* = 393)

From this study, the prevalence of immunological failure among HIV/TB co-infected patients was 48.8% (60/123). However, the prevalence of immune reconstitution failure among HIV mono-infected patients was 13.7% (37/270). The Study participants were failed immunologically as they got infected with TB (Fig. 1).

### Determinants of immune reconstitution to HAART among study participants

All variables with *p*-value less than 0.20 in the bivariate analysis were entered into a multivariate logistic regression model to assess the determinant factors for immune reconstitution failure. Multi collinearity and Hosmer Lemeshow goodness-of-fit test (0.058) were checked for each variable. Variables with a *p*-value less than 0.05 in the multivariate analysis were considered statistically significant at 95% confidence level. Finally, living in urban residence (AOR = 2.3, CI = 1.137–4.602, *p* = 0.020), having baseline CD4 count less than 250 (AOR = 4.2, CI = 2.997–7.961, *p* <  0.001), poor treatment adherence (AOR = 9.4, CI = 4.497–19.700, *p* <  0.001) and developing TB infection (AOR = 11.5, CI = 5.704–23.197, *p* <  0.001) were significantly associated with the immunological failure to HAART (Table 1).

## Discussion

In this study, the overall rate of immune reconstitution failure among HIV patients on HAART is 24.7% (97/393). This is similar to studies reported from Tanzania, 25% [26] and China, 18.4% [27]. However, studies from Northern Ethiopia, 6.5% [28], Liberia, 5.1% [29], Southern Ethiopia, 11.5% [30], Southwestern Ethiopia, 9.8% [31], Addis Ababa, 15.7 and 15% [32, 33], Northwestern Ethiopia 15.1% [34] and Colombia, 14% [35] reported lower immunological failure. To the contrary, other studies from Kenya, 64.4% [36], Thailand, 33.5% [37] and Nepal, 35% [38] reported higher immunological failure than this study. This variation in immunological response might be attributed to the differences in adherence to HAART. It might also be related to the WHO guideline which varies over time. In our study, we defined immunological failure, as the fall of CD4 + T cell count to baseline or below, or persistently low CD4+ T cell count (below 100 cells/μL) [6]. However, reports from the above studies [30–32, 35, 36] defined immunological failure as fall of CD4+ T cell count to baseline or below severe immune suppression (CD4+ T cell count < 200 cells/μL) [38], 50% fall from on-treatment peak value [33, 36] or 30% or above fall from on treatment peak value [26, 36]. Failure to achieve CD4+ T cell count above 350 cells/μL [27, 37].

In this study, the rate of immunological failure was 2.966 per 100 PY. This is in line with previous reports from Latin America, 2.57 per 100 PY [39]. However, the present study reported lower immune reconstitution failure than the studies from Northwestern Ethiopia, 8.7 per 100 PY [40] and Debremarkos, Ethiopia, 8 per 100 PY [41]. This variation might be due to the differences in adherence towards ART drugs, the WHO guidelines to define immunological failure/ success and the presence of opportunistic infections.

Two hundred eighty-five (72.5%) of our study participants had good adherence to treatment. Similar study reported from Southern Ethiopia, 81.8% [30], Northwestern Ethiopia, 82.7% [34], New Guinea, 82.4% [42] and Addis Ababa, Ethiopia, 78.5% [32]. However, our study reported lower treatment adherence than the studies conducted in Colombia, 92% [35]. Southern Ethiopia, 85.8% [30], Addis Ababa, Ethiopia, 97.7% [33] and Southwestern Ethiopia, 100% [31]. These variations in patient adherences might be due to differences in psychosocial support of relatives or the society, stigma and lack of commitment to take medications so that HIV patients might drop themselves from on course ART treatment, not feeling well (perceived it from the medication), scaring of treatment side effects and being busy (forgetting the HAART medication) [6, 43, 44].

The median baseline CD4+ T cell count of our study participants was 196 cells/μL. This is comparable with the studies reported in Southwestern Ethiopia, 191 cells/μL [31]. However, this is observed to be higher than the reports from Northern Ethiopia, 162 cells/μL [28], Southern Ethiopia, 156 cell/μL [30], Kenya, 152 cells/μL [36], Addis Ababa, 115 cells/μL [32] and 177 cells/μL [33]. A study from Liberia [29] reported a higher median baseline CD4+ T cell count of 238 cell/μL. This variation might be explained with the differences in time of HAART initiation among HIV patients for a long period. This is because a long duration of HIV infection without the ART leads to progressive viral replication, which in turn leads to lower CD4+ T cell count.

Lower baseline CD4+ T cell count (baseline CD4+ T cell count less than 250 cells/μL) was statistically associated with immune reconstitution failure of HAART (*p* <  0.001). This report is supported by previous studies from Debremarkos, Ethiopia [41] and Thailand [37]. Moreover, immune recovery depends on the baseline CD4+ T cell count. The timing of HAART initiation is important to optimize the CD4+ T cell immune response to medication [45]. These reports may highlight that patients with low CD4+ T cell count have poor long term CD4+ T cell immune response. Study participants with poor adherence towards HAART were 9.4 times more likely to experience CD4+ T cell recovery failure than those who had good adherence (*p* <  0.001). This is supported by a study conducted in Northwestern Ethiopia [34], Southern Ethiopia [36], Colombia [35] and France [46]. Poor treatment adherence might allow viral replication which in turn increases infection of more CD4+ T cells and ultimately depletion of their number [44].

This study revealed that, immune reconstitution failure was 11.5 times more likely to occur among TB co-infected individuals compared to TB non-infected individuals (*p* <  0.001). This was similar to reports from Southern Ethiopia [30], Gondar [40], and Nigeria [47]. TB infection impairs cellular immune responses through Mycobacterium tuberculosis-induced apoptosis of CD4+ T cells which subsequently lead to depletion of CD4+ T cells and results in immunological failure [48]. The prevalence of low immune reconstitution among HIV/TB co-infected participants was 48.8% (60/123) comparing to the failure of HIV mono-infected participants, 13.7% (37/270). The higher immune reconstitution failure among TB co-infected HIV participants is supported by previous studies from Uganda [49], South Africa [50] and Senegal [51]. This might be due to the fact that TB infection will contribute to the low CD+ T cells recovery by attaching and neutralizing CD4+ T cells. Incidence of TB during the course of HAART might have decreased the adherence f the treatment due to its high pill burden and side-effects [1].

The other associated factor for experiencing low immune reconstitution was residence. Participants from an urban residence were 2.1 times more likely to have low CD4+ T cells recovery than rural residents (*p* = 0.020). A previous study conducted in Ethiopia [52] has reported increased immunological failure among HIV patients living in urban areas. This might be due to the reason that HAART users of urban residence are more likely to be enrolled in harmful activities like chewing chat, smoking and drinking alcohol. Hence, experiencing immunological failure will be attributed to these possible factors.

## Conclusions

The overall rate of immune reconstitution failure among HIV patients on HAART was high. TB co-infection has shown to contribute to the higher rate of low CD4+ T cells to HIV therapy. Other factors like low CD4+ T cell baseline count, poor adherence and urban residence were also associated with low immune recovery. HIV patients who are coinfected with TB should be monitored for evaluation of CD4+ T cell count determination strictly. Hence, there would be a good immune recovery of HAART. Moreover, a virological response shall be assessed to HIV patients on HAART to determine the recovery from viral antigen replications.

## Data Availability

The datasets generated and/or analyzed during the current study are summarized in the manuscript but not publicly available due to confidentiality of patient results. However, the datasets can be shared from the corresponding author on reasonable request.

## Abbreviations

AOR: Adjusted odds ratio
ART: Antiretroviral Therapy
BMI: Body mass index
CI: Confidence interval
COR: Crude odds ratio
HAART: Highly Active Antiretroviral Therapy
HIV: Human immunodeficiency virus
TB: Tuberculosis
WHO: World health organization
